# Stat3 Is Important for Follicular Regulatory T Cell Differentiation

**DOI:** 10.1371/journal.pone.0155040

**Published:** 2016-05-05

**Authors:** Hao Wu, Markus M. Xie, Hong Liu, Alexander L. Dent

**Affiliations:** Department of Microbiology and Immunology, Indiana University School of Medicine, Indianapolis, Indiana, United States of America; Jackson Laboratory, UNITED STATES

## Abstract

The production of antibody is precisely controlled during the germinal center (GC) reaction. This process is dependent on the help from follicular T helper (Tfh) cells to germinal center (GC) B cells and is regulated by regulatory follicular T helper (Tfr) cells. How Tfr cells develop and how their suppressive activity functions are not well understood. Here, we found that Stat3 is indispensible for Tfr cell differentiation. After immunization with Sheep Red Blood Cells (SRBC), the loss of Tfr cells caused by deletion of Stat3 in Treg cells does not affect the size of Tfh or GC B cell population, but rather leads to strongly enhanced production of antigen-specific IgG1 and IgG2b. In Peyer’s patches (PPs) in the gut, we found that Stat3 expression in Treg cells is also required for Tfr cell formation to commensal organisms. However, loss of Tfr cells in the gut did not affect the numbers of Tfh cells and GC B cells, nor affect IgG1 or IgA switching by GC B cells. Overall, our study has uncovered unique roles of Stat3 in Tfr cell differentiation and the regulation of the antibody response.

## Introduction

To provide host protection against pathogens, CD4+ T cells differentiate into several distinct lineages that confer specific effector functions. For instance, viral or intracellular pathogens typically induce protective Th1 responses, while helminth parasites induce Th2 responses. Follicular T helper (Tfh) cells are a T helper cell lineage whose major function is to help B cells form germinal centers (GCs) and produce high-affinity antibodies (Abs) [1, 2]. Commitment of naïve T cells to these different effector lineages is highly dependent on the cytokines present in the immunological milieu. Cytokine receptor signaling activates specific transcription factor pathways, and the Stat4-Tbet, Stat6-Gata3 and Stat3-Rorγt pathways promote Th1, Th2 and Th17 cell differentiation respectively [3]. Tfh cells are driven by the transcription repressor Bcl6, which is induced downstream of Stat1, Stat3 or Stat4 activation [4–8]. Tfh cells control the initiation as well as the outcome of the GC B cell response. While Tfh cells are critical for the proper production of Abs, the over-production of Tfh cells can lead to autoimmunity, since Tfh cells can help B cells to produce self-reactive Abs [9–11]. Thus, the proper regulation of Tfh and GC B cell responses is essential both for normal immune function and for preventing autoimmune disease.

Foxp3 expressing regulatory T cells (Tregs) repress the activation, proliferation and function of effector T helper cells during immune responses and are critical to restrain autoimmune responses. In both humans and mice, deficiency of Foxp3 results in a fatal multi-organ autoimmune disorder. Specifically, Foxp3-mutant *Scurfy* mice have uncontrolled T cell proliferation and drastically elevated inflammation mediated by Th1, Th2 and Th17 responses as well as exacerbated production of autoreactive antibodies [12, 13]. Previous studies showed multiple subsets of Treg cells possess unique regulatory properties to repress corresponding pathological immune responses. For example, Tbet controls Treg functions during Th1 mediated inflammation [14]. IRF4 in Treg cells is important for regulation of Th2 responses [15]. Deletion of Stat3 in Treg cells results in dysregulation of Th17 responses [16–18]. More recently, a subpopulation of Foxp3+ Treg cells has been found with Tfh-like properties that appears to act as suppressors of the GC response [19–21]. These regulatory follicular T cells or “Tfr” cells depend on Bcl6 for differentiation and chemokine receptor CXCR5 to localize to the B cell follicle and the GC [19–21].

Ding *et al* revealed that IL-21, which activates Stat3 signaling, can suppress Tfr cell differentiation in BXD2 autoimmune mice [22]. At the same time, IL-6-Stat3 and IL-21-Stat3 signaling can promote Tfh cell differentiation by induction of Bcl6 expression [6, 8, 23]. It is therefore important to determine whether Stat3 promotes or inhibits Tfr cell development. Treg cells tend to adopt part of the transcriptional program of the specific T helper cell subsets they regulate. Therefore, the requirement for Stat3 in Treg cells to suppress Tfh cell mediated humoral responses is of particular interest. Previous studies show that in Peyer’s patches (PPs), Tfr cells, Tfh cells and Th17 cells all function to promote the production of IgA antibodies, which in turn act to maintain bacterial diversity at the mucosal barrier in the gut [24, 25]. However, the role of Stat3 in the regulation of gut immune responses by Treg cells is not clear.

Here, we found that Stat3 is essential for Tfr cell differentiation both in spleen following antigen immunization and in PPs. Deletion of Stat3 causes a much more severe defect in Tfr cell differentiation than in Tfh cell differentiation. Interestingly, loss of Tfr cells in Stat3^fl/fl^Foxp3^cre^ mice has no gross effect on the size of Tfh or GC B cell population after immunization, but does lead to increased production of antigen-specific IgG antibodies. In addition, Stat3 function in Treg cells is not required for normal levels of IgA and IgG1 class switching in GC B cells in the gut.

## Materials and Methods

### Mice and immunizations

Stat3^fl/fl^ mice were crossed with CD4-cre transgenic mice or Foxp3-YFP-cre mice. Bcl6^fl/fl^ mice were crossed with Foxp3-YFP-cre mice. Foxp3-YFP-cre mice and B6.SJL-PrprcaPepcb/BoyJ (BoyJ) mice were purchased from The Jackson Laboratory (JAX). For Stat3^fl/fl^CD4^cre^ (Stat3CD4KO) mice, littermate Stat3^fl/fl^CD4^+^ mice were used as WT control. For Stat3^fl/fl^Foxp3^cre^ (Stat3FC), littermate Foxp3^cre^ mice were used as WT control. Mice were bred under specific pathogen-free conditions at the laboratory animal facility at Indiana University School of Medicine (IUSM), and were handled according to protocols approved by the IUSM Animal Use and Care Committee (IACUC). The animal facility carries the NIH animal welfare assurance number A4091-01. Mice were not subjected to any pain or distress greater than injection, and all efforts were taken to minimize animal suffering. Mice were euthanized by CO_2_ asphyxiation, the preferred method by our internal review board. For sheep red blood cell (SRBC) immunization, mice were intraperitoneally (i.p.) injected with 1 x 10^9^ SRBCs (Rockland Immunochemicals) and were sacrificed at the indicated day.

### Flow cytometry reagents

Anti-CXCR5 (2G8) and GL7 (GL7) Abs were from BD Biosciences. Fixable viability dye, anti-CD38 and anti-Foxp3 (FJK-16s) Abs were from eBioscience. Anti-CD4 (GK1.5), anti-B220 (RA3-6B2), anti-CD45.1 (A20), anti-PD-1 (29F.1A12), anti-IgG1 (RMG1-1) and anti-IgA (RMA1) were from Biolegend.

### Cell staining for flow cytometry

After red blood cell lysis, total spleen cells were incubated with anti-mouse CD16/CD32 (BioXcell) for 5 minutes at RT, followed by surface staining for the indicated markers. For intracellular transcription factor staining, after surface markers were stained, cells were fixed and stained with antibodies against transcription factors by following Foxp3 fixation kit (eBioscience) instructions. Cell events were collected on an LSRII flow cytometer (Becton Dickonson).

### Peyer’s patch (PP) isolation

PP’s were cut using scissors from the small intestine and incubated for 10 mins at 37°C in PBS containing 1% FBS, 4mM EDTA and 15 mM HEPES (PH 7.2). PP’s were washed twice, with vigorous vortexing before spinning, in PBS. Soluble cell debris in supernatants was removed after centrifugation. Isolated PPs were broken apart between two frosted glass microscope slides to generate single cell suspension for flow cytometry staining or ICS described above.

### Bone marrow chimeras

Recipient CD45.1+ BoyJ mice were lethally irradiated with 1,100 Rad and reconstituted with 2x10^6^ a mixture of nucleated bone marrow cells from CD45.2+ WT mice plus CD45.1+ BoyJ mice, or CD45.2+ Stat3CD4KO mice plus CD45.1+ BoyJ mice by i.v. injection. Chimeric mice were immunized with SRBC approximately 12 weeks after reconstitution.

### Antibody titer analysis

Antibody titers of SRBC-specific Ig and total Ig in serum were measured by ELISA, similar to previously reported [26]. For anti-SRBC Ab analysis, 96 well Nunc-Immuno plates were coated with SRBC membrane protein overnight at 4°C. For anti-mouse IgA, anti-mouse total IgG or anti-mouse IgM Abs, directly peroxidase-conjugated secondary Abs (Sigma-Aldrich) were used. For analysis of IgG1 (A85-1, BD Biosciences), IgG2b (R12-3, BD Biosciences), IgG2c (Abcam) and IgG3 (R40-82, BD Biosciences), biotin-labeled secondary Abs were used along with Avidin-HRP (BD Biosciences). For total Ig isotype analysis, 96 well Nunc-Immuno plates were coated with anti-Ig kappa light chain Ab (187.1, BD Biosciences) diluted in PBS overnight at 4°C. Wells were blocked with 10% FCS and diluted serum was added and incubated at room temperature for 2 h.

### Statistical Analysis

All data analysis was done using Prism Graphpad software. Unless otherwise stated, Student t test or ANOVA with Tukey post hoc analysis were used. Only significant differences (*p* < 0.05) are indicated in figures.

## Results

### Stat3 deficiency in T cells results in a decrease of Tfh cells and a block of Tfr cell differentiation

**Fig 1:**
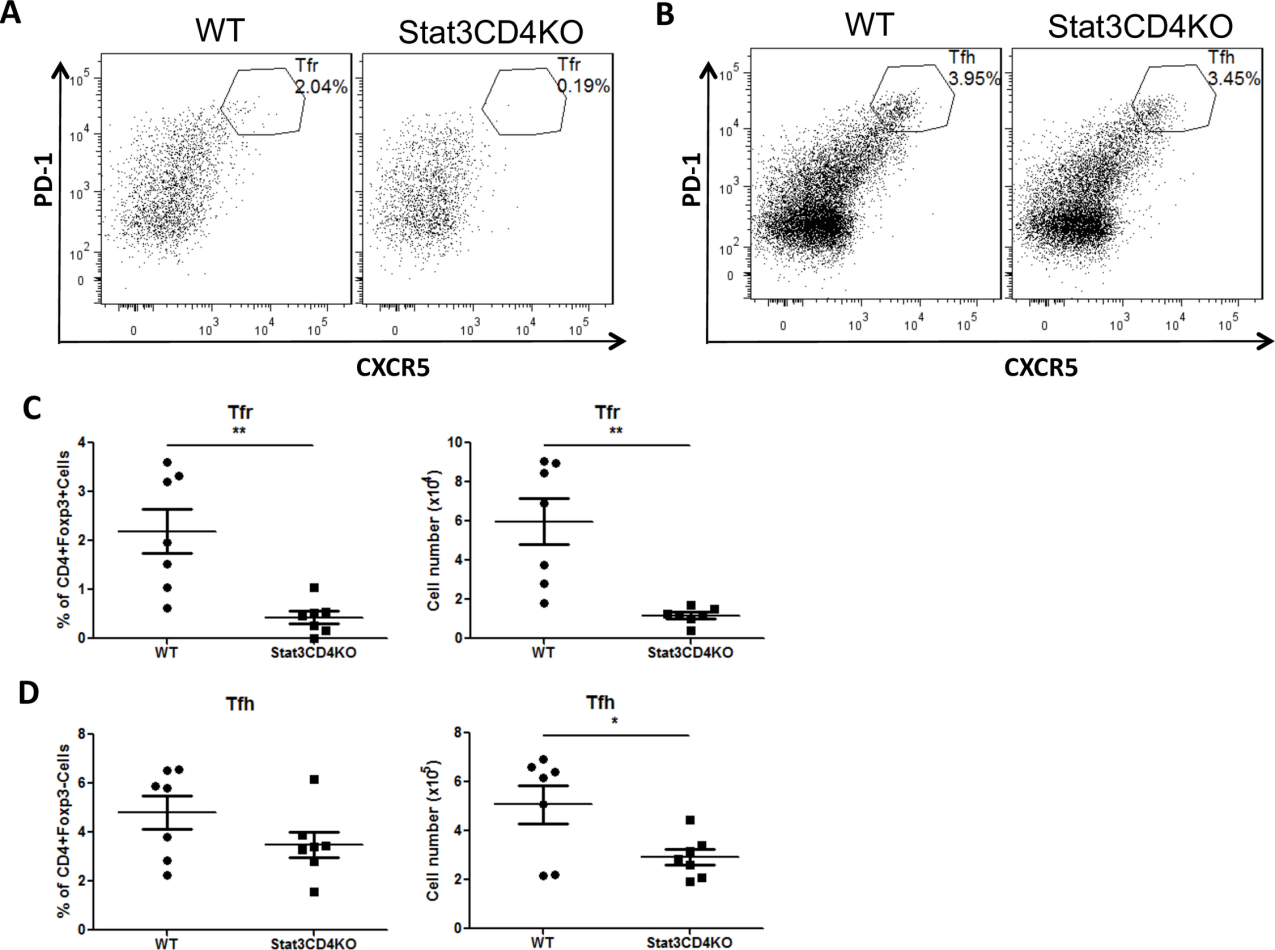
Stat3CD4KO mice have impaired Tfh and Tfr cell responses following sheep red blood cell (SRBC) immunization. Control and Stat3CD4KO mice were immunized with SRBC via i.p. injection. Spleens were isolated for flow cytometric analysis at 7 days post immunization (dpi). (A and B) Flow cytometry plots of Tfr cells which are CD4+Foxp3+CXCR5^hi^PD-1^hi^ (A) and Tfh cells which are CD4+Foxp3-CXCR5^hi^PD-1^hi^ (B). (C) Tfr cells percentages (left) and numbers (right) gated as in (A). (D) Tfh cells percentages (left) and numbers (right) gated as in (B). Graphs show mean +/- SEM, n = 7. *p < 0.05, **p < 0.01, *** *p* < 0.001 (student *t* test). Data are combined from two independent experiments.

To test whether Stat3 is required for the differentiation of Tfr cells, we immunized control and Stat3CD4KO mice with SRBC and found that loss of Stat3 severely blocked the differentiation of Foxp3^+^CXCR5^hi^PD1^hi^ Tfr cells (Fig 1A and 1C). Consistent with our previous results [26], in Stat3CD4KO mice, deletion of Stat3 leads to a decrease of Foxp3^-^CXCR5^hi^PD1^hi^ Tfh cell in cell number, but not in percentage (Fig 1B and 1D). Both Tfh cells and Tfr cells were significantly decreased, however Tfr cells were almost gone whereas 60% of Tfh cells still remained in the absence of Stat3 in T cells. We calculated the relative decrease levels for Tfh and Tfr cells due to deletion of Stat3, and found that the average loss of Tfr cells (>80% decrease) is much higher than the average loss for Tfh cells (~40% decrease). These results suggest that Stat3 in CD4 T cells are required for normal Tfh and Tfr cell differentiation, but that Tfr cells are more dependent on Stat3 than Tfh cells.

### The differentiation defect in Tfr cell differentiation is Treg cell intrinsic

**Fig 2:**
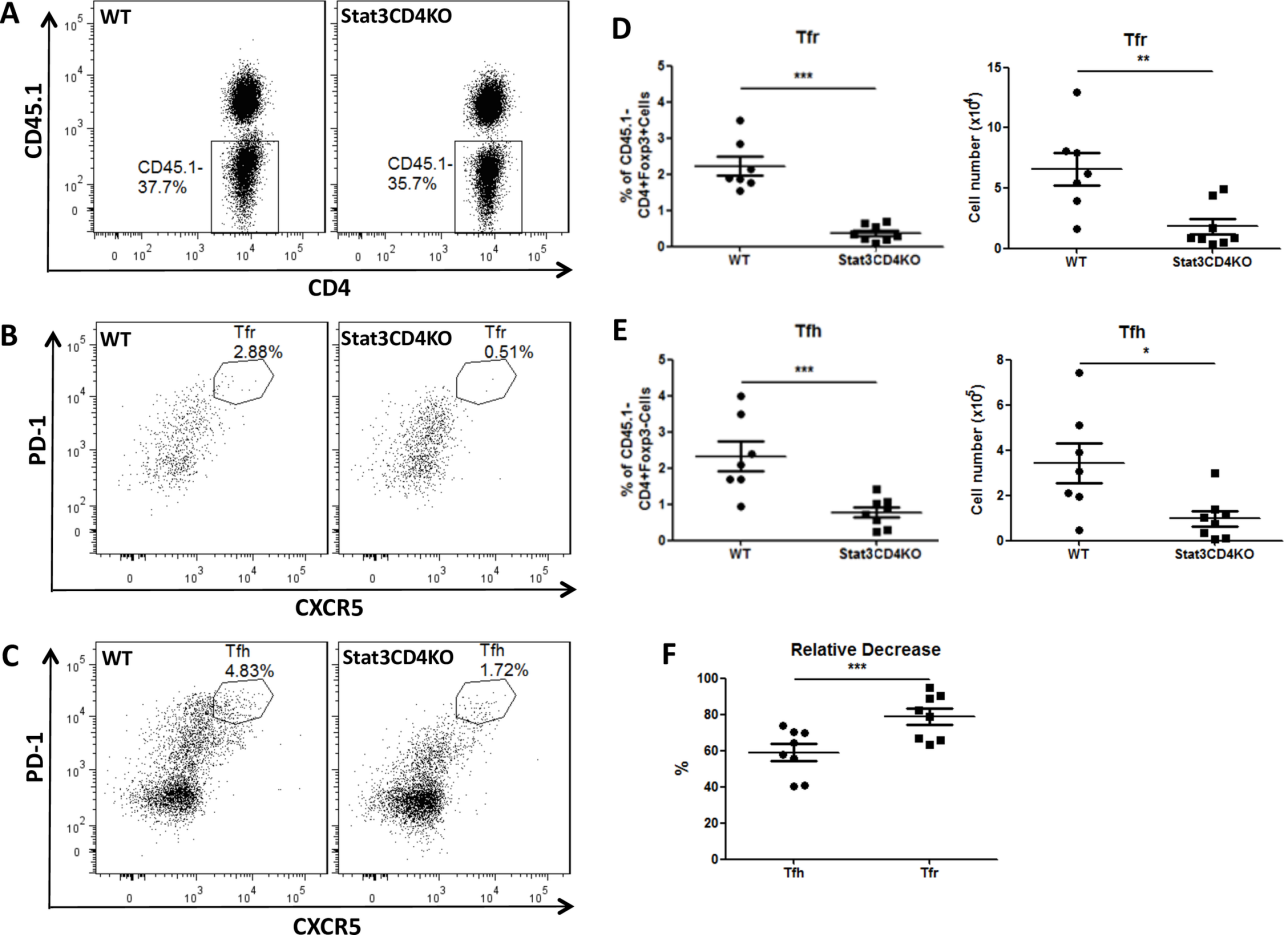
Intrinsic defect of Stat3-deficient Tfh and Tfr cell differentiation. (A-F) CD45.1^-^ bone marrow (BM) cells from WT or Stat3CD4KO mice mixed with CD45.1^+^ BM cells from BoyJ mice were transplanted into lethally irradiated BoyJ mice. After 3 months, chimera mice were immunized with SRBC via i.p. injection. Spleens were isolated for flow cytometric analysis at 7 dpi. (A) Flow cytometry plots of splenic CD45.1^-^CD4^+^ cells derived from WT or Stat3CD4KO donor BM. (B) Flow cytometry plots of Tfr cells which are CD4+Foxp3+CXCR5^hi^PD-1^hi^ generated from WT (left) and Stat3CD4KO (right) donor BM. (C) Flow cytometry plots of Tfh cells which are CD4+Foxp3-CXCR5^hi^PD-1^hi^ generated from WT (left) and Stat3CD4KO (right) donor BM. (D) Tfr and (E) Tfh cells percentages (left) and numbers (right) gated as in (B) and (C) respectively. Graphs show mean +/- SEM, n = 7–8. *** *p* < 0.001 (student *t* test). Data are combined from two independent experiments.

The population of GC B cells was dramatically decreased in Stat3CD4KO mice [26] and GC B cells are important for Tfh cell differentiation and maintenance [27]. Tfr cells may be similarly dependent on B cells, and the loss of Tfr cells in Stat3CD4KO mice could be due to either lack of GC B cell support or loss of intrinsic Stat3 function in the Treg cells. To investigate these possibilities, we generated mixed BM chimeras in which we transferred CD45.1- BM donor cells from WT or Stat3CD4KO mice mixed with CD45.1+ BM donor cells from BoyJ mice into lethally irradiated CD45.1+ BoyJ mice. After 3 months of engraftment (Fig 2A), we immunized the chimera mice with SRBC and analyzed spleen cells at 7 days post immunization (dpi). Both mice that received WT BM and Stat3CD4KO BM had similar populations of GC B cells (data not shown). Whereas WT donor cells were able to differentiate into Tfr and Tfh cells, Stat3CD4KO donor cells had a severe defect in Tfr and Tfh cells (Fig 2B–2E). The decrease of Tfr cells (~80%) was much higher than of Tfh cells (~60%) in the chimera mice, consistent with the results from the directly immunized WT and Stat3CD4KO mice (Fig 1). These results suggest that Tfr cell differentiation intrinsically requires Stat3, and that Tfr cell differentiation is more dependent on Stat3 signaling than Tfh cell differentiation.

### Loss of Tfr cells does not lead to an increased Tfh cell population in Stat3FC mice

**Fig 3:**
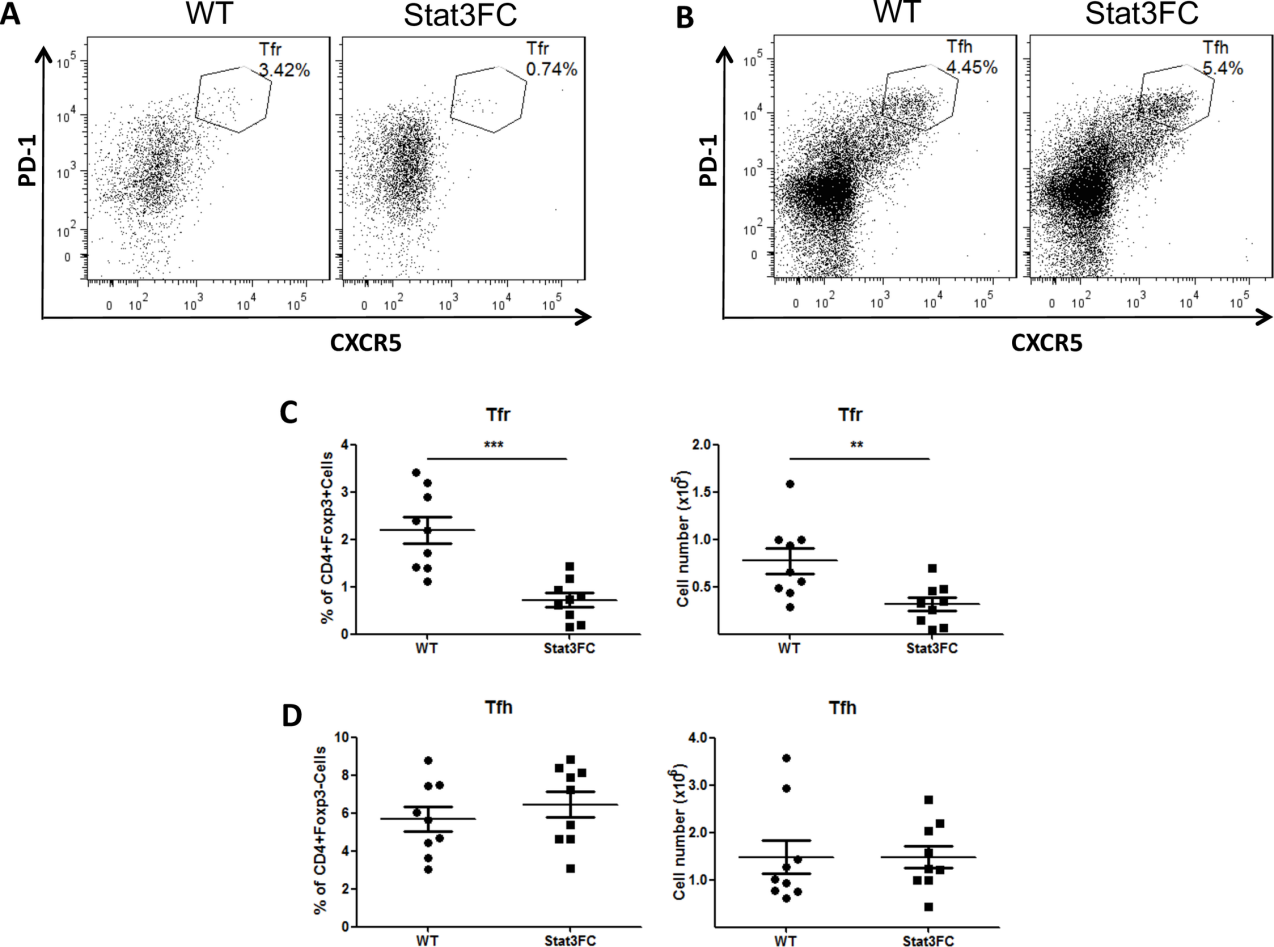
Loss of Tfr cells in Stat3FC mice does not affect the size of Tfh cell population after SRBC immunization. Control and Stat3FC mice were immunized with SRBC via i.p. injection. Spleens were isolated for flow cytometric analysis at 10 dpi. (A and B) Flow cytometry plots of Tfr cells, which are CD4+Foxp3+CXCR5^hi^PD-1^hi^ (A) and Tfh cells which are CD4+Foxp3-CXCR5^hi^PD-1^hi^ (B). (C) Tfr cells percentages (left) and numbers (right) gated as in (A). (D) Tfh cells percentages (left) and numbers (right) gated as in (B). Graphs show mean +/- SEM, n = 9. ** *p* < 0.01, *** *p* < 0.001 (student *t* test). Data are combined from three independent experiments.

In order to assess the functional role of Stat3 in Treg cells regulating GC response, we created Stat3^fl/fl^Foxp3^cre^ (Stat3FC) mice, where floxed Stat3 allele is deleted specifically in Foxp3 expressing T cells. In contrast to what was reported in a previous study [16], Stat3FC mice are healthy, fertile, do not display abnormal inflammation and have grossly normal immune cell compartments up to at least 5 months of age (data not shown). We immunized WT and Stat3FC mice with SRBC and analyzed Tfh and Tfr cells in spleen at 10 dpi. As expected, the differentiation of Foxp3^+^CXCR5^hi^PD-1^hi^ Tfr cells was strongly diminished in Stat3FC mice (Fig 3A and 3C). We did not see an increase in the proportion or number of Tfh cells in immunized Stat3FC mice (Fig 3B and 3D). This result is not unique to SRBC immunization, as Stat3FC mice also had comparable Tfh cell population as control mice did at 25 dpi of SRBC immunization as well as following KLH-Alum immunization (data not shown). We also found a similar phenotype in Bcl6^fl/fl^Foxp3^cre^ (Bcl6FC) mice, where Bcl6 is specifically deleted in Treg cells [28]. Bcl6FC mice had a severe depletion of Tfr cells, however they had normal Tfh cell differentiation in spleen after immunization [28]. Thus, while Tfr cells are strongly decreased in both Stat3FC and Bcl6FC mice, loss of Tfr cells does not necessarily lead to an enlarged Tfh cell population.

### Loss of Stat3-dependent Tfr cells does not lead to an increased GC B cell population but increases antigen-specific IgG antibodies

**Fig 4:**
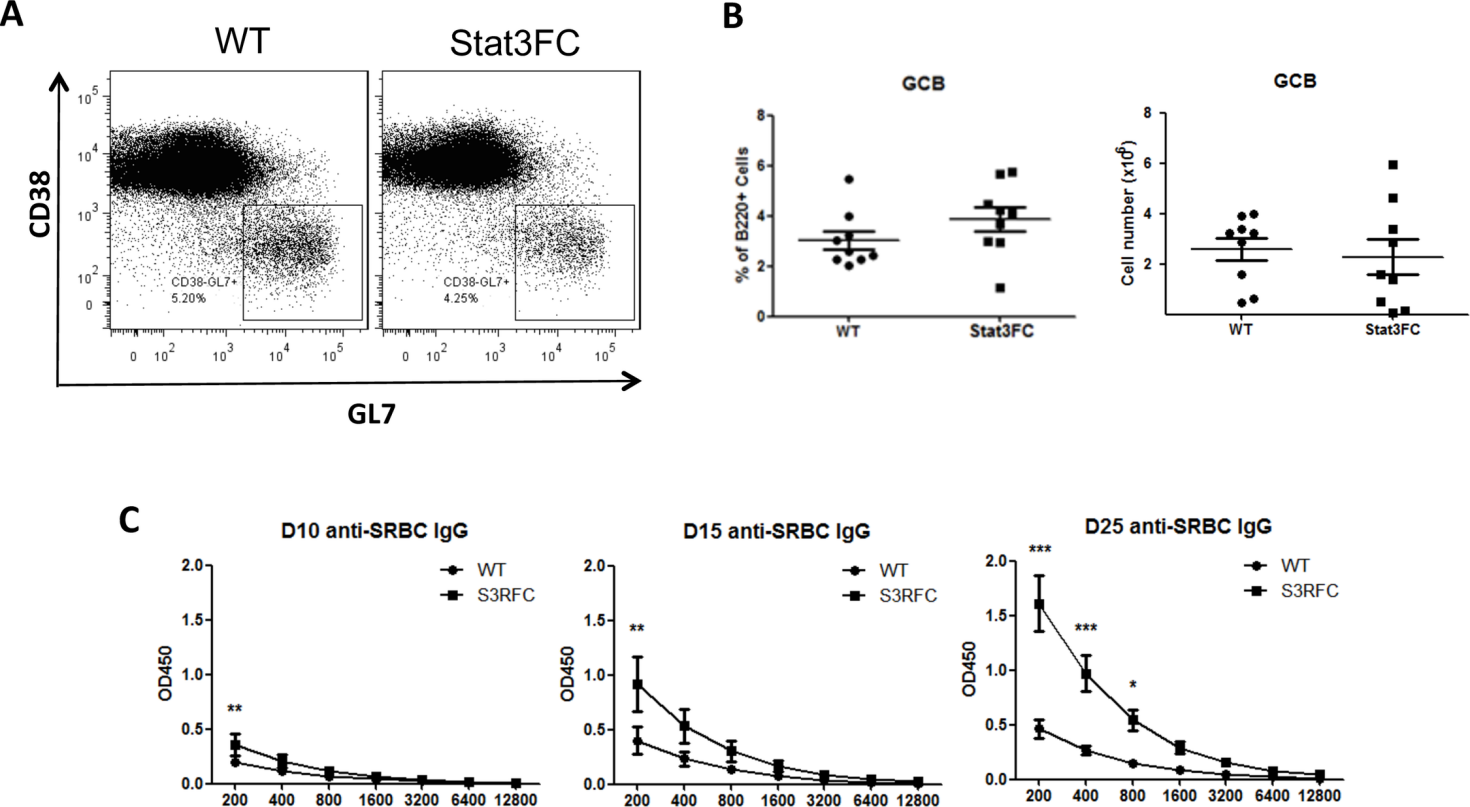
Loss of Tfr cells in Stat3FC mice does not affect the size of GC B cell population but increases the anti-SRBC IgG titers after SRBC immunization. Control and Stat3FC mice were immunized with SRBC via i.p. injection. Spleens were isolated for flow cytometric analysis at 10 dpi. (A) Flow cytometry plots of GC B cells, which are B220+CD38-GL7+. (B) GC B cells percentages (left) and numbers (right) gated as in (A). (C) Control and Stat3FC mice were immunized with SRBC. Serum samples were collected at 10, 15 and 25 dpi. anti-SRBC IgG titers are shown. The X-axis shows the dilution factors. Graphs show mean +/- SEM, (B) n = 9, (C) n = 5. ** *p* < 0.01, *** *p* < 0.001 (student *t* test for B, two-way ANOVA for C). For B, data are combined from three independent experiments. For C, data are representative of three independent experiments with similar results.

**Fig 5:**
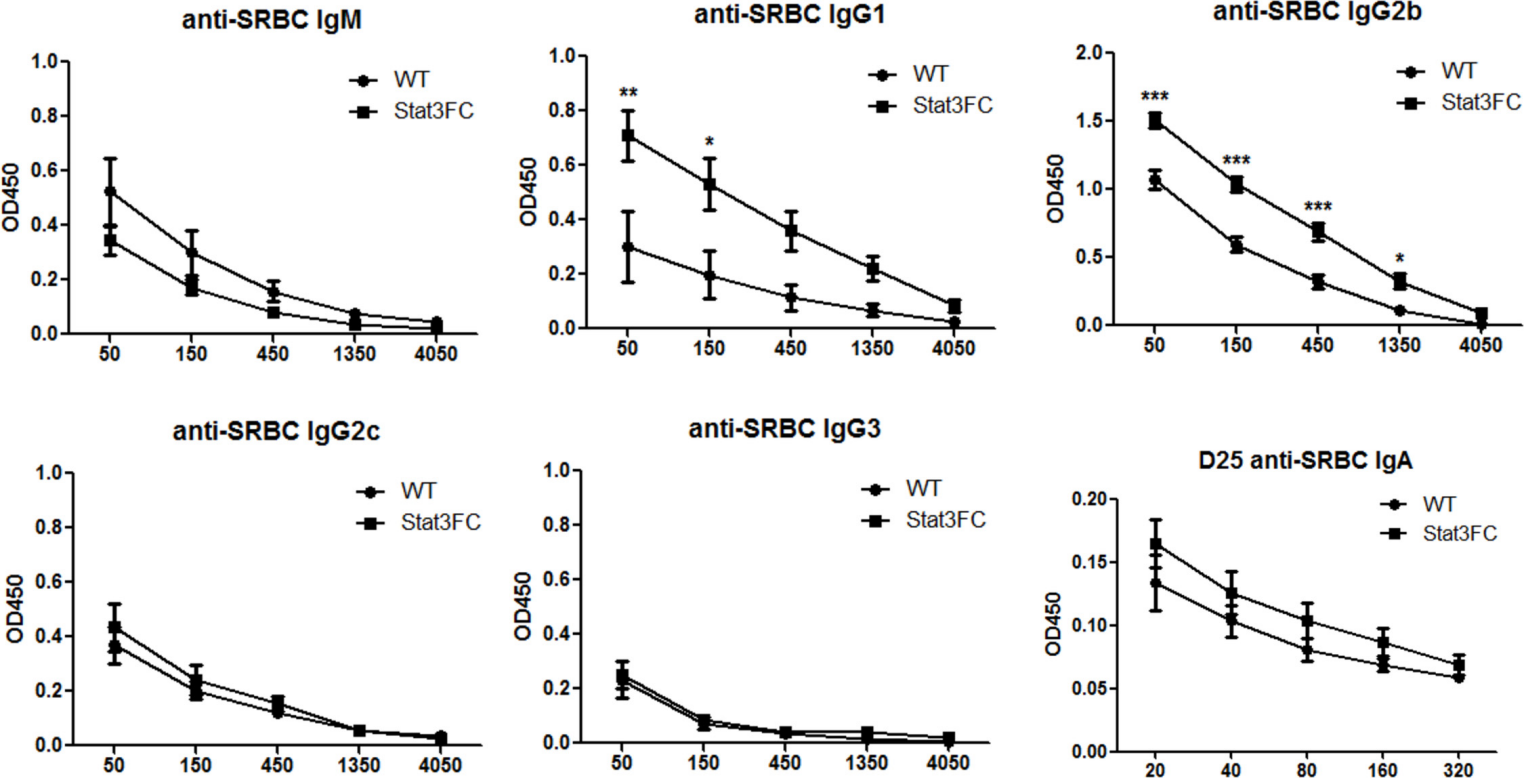
Tfr cells regulate the production of anti-SRBC IgG1 and anti-SRBC IgG2b. Control and Stat3FC mice were immunized with SRBC via i.p. injection. Serum samples were collected at 25 dpi. Anti-SRBC IgM, IgG1, IgG2b, IgG2c, IgG3 and IgA titers are shown. SRBC-specific IgD and IgE were not detectable in the serum. The X-axis shows the dilution factors. Graphs show mean +/- SEM, n = 4. ** p < 0.01, *** p < 0.001 (two-way ANOVA). Data are representative of two independent experiments with similar results.

**Fig 6:**
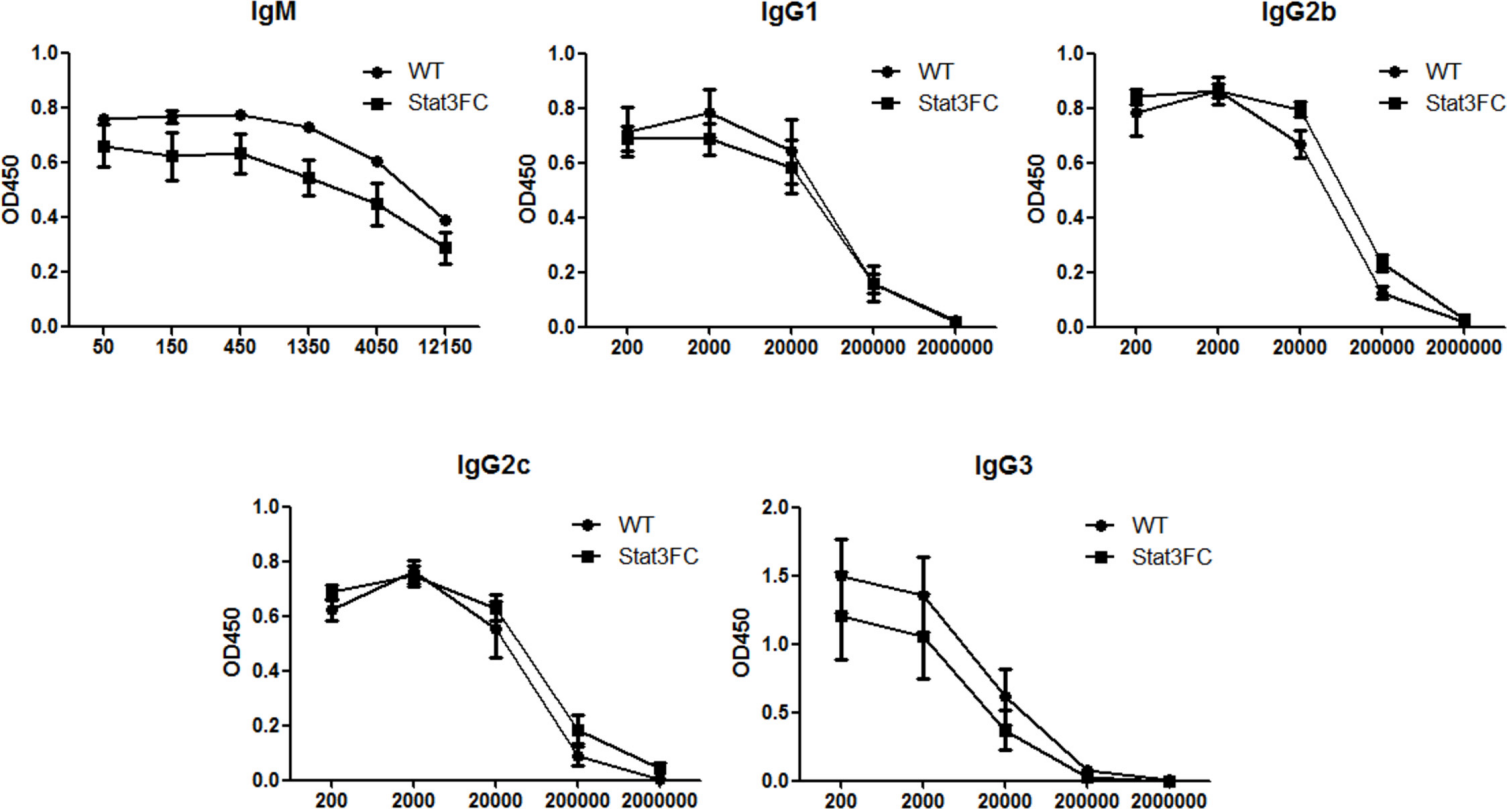
Basal titers of serum IgM, IgG1, IgG2b, IgG2c and IgG3 are not altered in Stat3FC mice. Serum samples from unimmunized control and Stat3FC mice were collected. IgM, IgG1, IgG2b, IgG2c and IgG3 titers are shown. The X-axis shows the dilution factors. Graphs show mean +/- SEM, n = 3. No significant differences in basal Ig isotype levels were observed by two-way ANOVA. Data are representative of two independent experiments with similar results.

Previous reports show Tfr cells can directly inhibit B cell responses [19–21]. We next wondered whether this is the case in our Stat3FC mice. Interestingly, at 10 dpi, similar sizes of B220^+^CD38^-^GL7^+^ GC B cell populations were formed both in control and Stat3FC mice (Fig 4A and 4B). Similar to our observations with Tfh cells, this result of unchanged GC B cell numbers in the absence of Tfr cells was observed at 25 dpi for both SRBC and KLH-Alum immunization (data not shown). This is again consistent with our data from Bcl6FC mice [28]. Since the outcome of GC response is the generation of high-affinity antigen-specific isotype-switched antibodies, we next analyzed SRBC-specific IgG serum antibody titers at 10, 15 and 25 dpi by ELISA. We found that, at all the three time points, the titers of anti-SRBC IgG antibodies were significantly increased in Stat3FC mice. The magnitude of the increase in Ab titer for Stat3FC mice became larger over time, and the anti-SRBC IgG antibody titer at 25 dpi showed the largest difference (Fig 4C). These results indicate that Stat3 is required for Treg/Tfr cells to suppress antigen-specific antibody production. We next analyzed the specific subtypes of IgG Abs increased in Stat3FC mice, as well as whether other types of Abs are controlled by Stat3-dependent Tfr cells. As shown in Fig 5, the specific IgG subtypes increased in the absence of Stat3-dependent Tfr cells are IgG1 and IgG2b. SRBC-specific IgG2c and IgG3 Abs were detected, but were not increased in Stat3FC mice (Fig 5). SRBC-specific IgM and IgA responses were elevated in Stat3FC mice but the increase was not significant (Fig 5). SRBC-specific IgD and IgE responses were not detected in either WT or Stat3FC mice (data not shown). We additionally assessed whether Stat3-dependent Tfr cells controlled basal levels of Ig secretion. As shown in Fig 6, basal levels of IgM and IgG subtypes were essentially normal in Stat3FC mice. A similar finding was seen with Stat3CD4KO mice (S1 Fig), supporting the idea that neither Tfh cells nor Tfr cells control the basal levels of IgM and IgG subtypes.

### Stat3 function in Treg cells is also required for Tfr cell differentiation in Peyer’s patches

**Fig 7:**
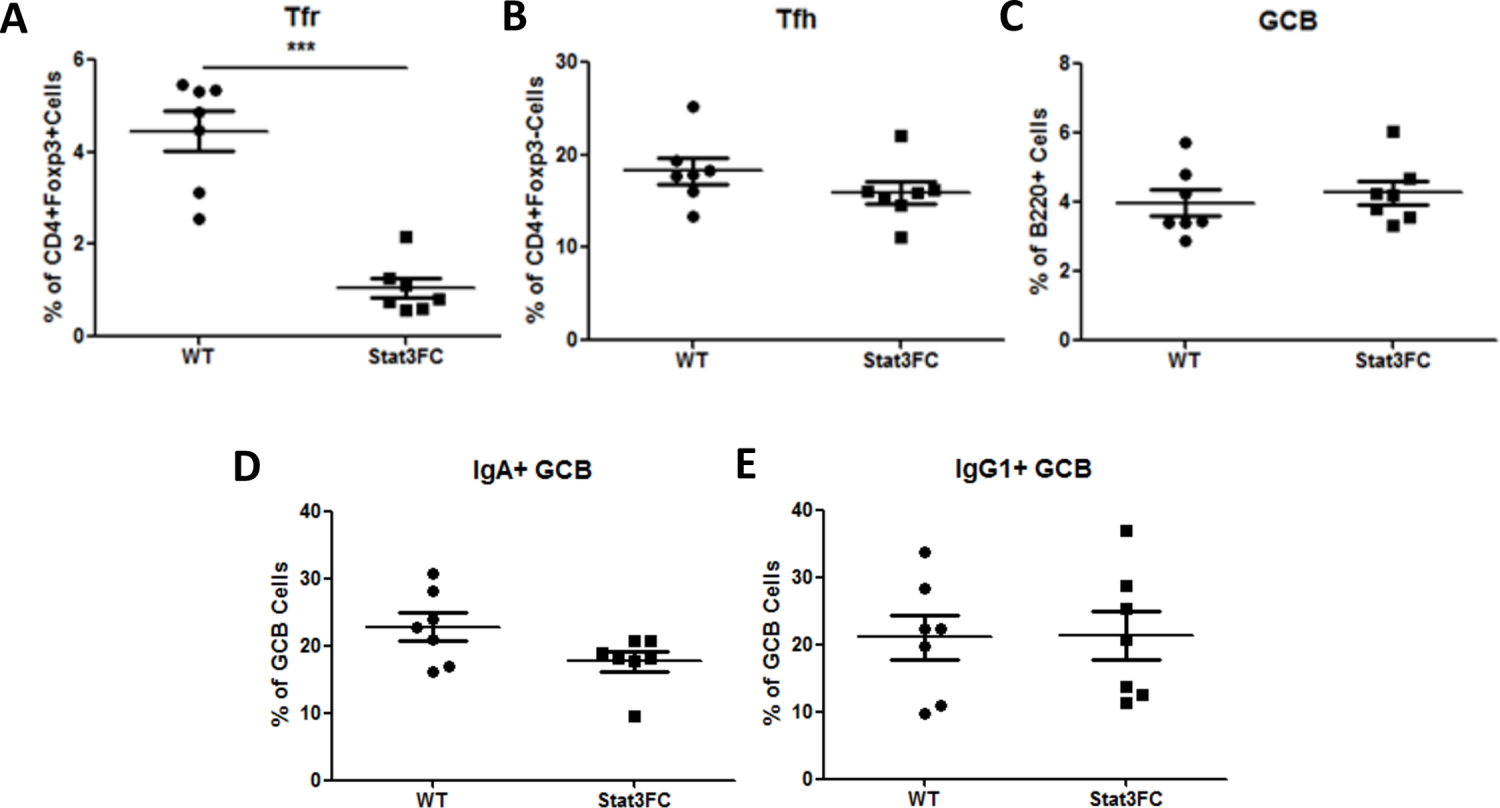
In Peyer’s patches, Treg cell specific Stat3 deletion leads to the loss of Tfr cells and does not have an effect on Tfh, GC B cells, as well as IgG1+ and IgA+ GC B cells. PPs from control and Stat3FC mice were isolated and homogenized into single cell suspensions for flow cytometric analysis. The percentages of Tfr cells (A), Tfh cells (B), GC B cells (C), IgA+ GC B cells (D) and IgG1+ GC B cells (E) are shown. Graphs show mean +/- SEM, n = 7. *** *p* < 0.001 (student *t* test). Data are combined from two independent experiments.

A previous study showed that Treg cells can differentiate into Tfh cells in PPs in the small intestine [29]. We found that Stat3 is required for both Tfh and Tfr cell differentiation in spleen (Figs 1C, 1D, 2D and 2E). To further investigate the role of Stat3 in Treg cells in PPs, we examined Tfr and Tfh cell differentiation in PPs in Stat3FC mice. In Stat3FC mice, Tfr cell differentiation was severely decreased, but Tfh cell and GC B cell differentiation was left intact (Fig 7A–7C). These results are unchanged when absolute numbers of these cell populations are analyzed (data not shown). GC B cells switch to IgG1 and IgA at high rates in the PP, and we were able to detect about 20% of PP GC B cells that expressed IgG1 and about 20% that expressed IgA (Fig 7D and 7E). However, switching to IgG1 and IgA in the PP was not perturbed by the loss of Tfr cells in Stat3FC mice (Fig 7D and 7E). Overall, these results indicate that Stat3 is required for Tfr formation in the PP, but Stat3-dependent Tfr cells do not grossly affect Tfh or GC B cell numbers or isotype class switching in the PP. Our data show that Tfr cells are almost completely Stat3-dependent for their formation, but that Stat3-dependent Tfr cells have little impact on Tfh and GC B cell proliferation. Overall, Stat3-dependent Tfr cells have relatively subtle roles in fine-tuning the GC and Ab response.

## Discussion

In this study, we found that Stat3 is required for Tfr cell differentiation in spleen after antigen immunization as well as in PPs that receive constitutive antigen stimulation from gut microbiota. Mice with deletion of Stat3 in Treg cells still have normal differentiation of Tfh and GC B cells, however the production of antigen-specific IgG1 and IgG2b antibodies is strongly increased in these mice. In addition, switching to IgA and IgG1 in PP GC B cells was not affected by the loss of Tfr cells due to Treg-specific Stat3 deletion. Thus, our study has unveiled an essential role for Stat3 in Tfr cell differentiation as well as revealed the complex regulatory function of Tfr cells on antibody production.

Treg cells and effector T cells in a given immune response experience the same immunological microenvironment including cytokine signals, and therefore, similar to effector T cells, Treg cells express certain lineage determining transcription factors, like Bcl6, T-bet, IRF4 and Stat3 [30], to promote their lineage-specific suppression function. For Tfr and Tfh cells, they share similar differentiation process, activated by dendritic cells (DCs) in the T cells zone and maintained by B cells in the GC [31]. They express similar signature genes, CXCR5, PD-1, ICOS and Bcl6, which indicates that similar upstream genes or signaling might be involved in both Tfr and Tfh cell differentiation. Since the T cell zone and the GC are rich in IL-6 and IL-21, Stat3 signaling is one of the potential lineage determining pathways shared by Tfh cells and Tfr cells. Here we found that, in both an antigen immunization model and in PPs, Stat3 is indispensible for Tfr cell differentiation. In Ding *et al* [22], they showed that IL-21 can suppress Tfr cell differentiation while promoting Tfh cell differentiation in BXD2 autoimmune mice. However, since Stat1 and Stat3 can be downstream of IL-21 receptor signaling [32], Stat1 may inhibit the differentiation of Tfr cells while Stat3 promotes Tfr differentiation. Another possibility is that chronic inflammation in BXD2 mice causes the expansion of Tfh but not Tfr cells, and thus IL-21 does not truly inhibit Tfr cell differentiation. Of note, our data from chimeric mice suggest that Tfr cells are intrinsically more dependent on Stat3 signaling for differentiation than Tfh cells. Our data indicate that in Stat3-deficient Foxp3– CD4 cells, Bcl6 still can be up-regulated by Stat1 or Stat4 to allow significant Tfh cell differentiation. However, in Stat3-deficient Treg cells, Stat1 or Stat4 cannot compensate the Tfr cell differentiation defect in the absence of Stat3.

Chaudhry *et al* reported that Treg cell specific deletion of Stat3 in mice can lead to severe autoimmune diseases in multiple organs by 12–14 weeks of age [16]. However, similar to what was observed with Kluger et al [17, 18], our Stat3FC mice are healthy, fertile, have normal sized lymphoid organs and do not display abnormal inflammation up to 5 months of age. One difference with our work and Chaudry et al is that we use different strains of Stat3^fl/fl^ mice. Exons 16–21 of Stat3 locus are floxed in our Stat3^fl/fl^ mice [33], whereas exons 21–22 are floxed in Chaudhry *et al*’s Stat3^fl/fl^ mice [34]. However, both types of Stat3 deletions lead to complete loss of Stat3 function [33, 34]. Thus, a likely explanation is that different housing conditions, differences in specific pathogen-free conditions or differences in endogenous flora in the animal facilities lead to phenotypic differences for similar strains of mutant mice [35].

Our data here with Stat3FC mice helps explain some of the findings of Kluger *et al*, who used similar mice with conditional deletion of Stat3 by Foxp3-cre to investigate autoimmune kidney disease [17, 18]. For ease of discussion, we will also term these Kluger *et al* conditional Stat3 knockout mice as “Stat3FC”. In one study with these mice, crescentic glomerulonephritis (CGN) was induced by injection of nephrotoxic sheep serum [17]. In this study, higher levels of disease were seen in the Stat3FC mice, along with elevated levels of sheep Ig-reactive IgG2b and IgG2c. Our data presented here indicate that these increased IgG responses were likely due to the loss of Tfr cells. Interestingly, IgG2b was elevated in Stat3FC mice in both our study and the sheep serum study [17], and IgG2b switching is known to be promoted by TGF-beta signaling [36]. Increased TGF-beta was observed in the Stat3FC mice by Kluger et al [17], which may explain the increased IgG2b responses in these mice. In a second study, pristane was used to induce lupus-like nephritis [18]. In contrast to the CGN study [17], auto-immune Abs induced by pristane were similar or significantly lower in StatFC mice compared to controls, despite increased kidney disease, increased Th17 responses and greatly increased death from pulmonary vasculitis in the Stat3FC mice [18]. The question then arises: why weren’t increased IgG responses seen in the pristane model? We have data that the repressive function of Stat3-dependent Tfr cells on IgG responses can depend on the type of antigen used. We tested an HIV gp120 prime-boost immunization model in the Stat3FC mice but observed no differences in overall anti-gp120 IgG titer or affinity (data not shown). The HIV gp120 immunization model involves multiple injections over several months [28, 37], and like pristane injection, is more like a chronic immune response than an acute immunization response. Thus, Stat3-dependent Tfr cells may play a more critical role in limiting Ig production during acute immune responses than in chronic immune responses. We should note that we attempted to induce auto-Abs in our StatFC mice with pristane, but observed pervasive and fatal toxicity shortly after injection (data not shown). This result may be related to the specific C57BL/6 inbred mouse strain background we used for the Stat3FC mice.

Other studies on Tfr cells have observed increased Tfh and GC B cell responses in the absence of Tfr cells [19–21]. However, our Stat3FC mice have normal sizes of Tfh and GC B cell populations both in spleen after immunization and in PPs. Our findings here showing limited Tfr cell effects on Tfh and GC B cells are consistent with a separate study of Tfr cell function we conducted using Bcl6FC mice. Bcl6FC mice also have a severe loss of Tfr cells yet have similar sizes of Tfh and GC B cell populations compared to their WT controls [28]. One possibility is that the non-physiological conditions caused by T cell transfer into T cell deficient mice, and the non-specific effects of total Treg cell depletion led to the enhanced Tfh and GC B cell responses observed in earlier studies of Tfr cells.

With Stat3FC mice, we see increased production of SRBC-specific IgG antibodies, without a change in SRBC-specific IgA. This contrasts with Bcl6FC mice, where we observed decreased SRBC-specific IgG and increased SRBC-specific IgA along with decreased Tfr cells [28]. The reason for this difference is not yet clear, but likely relates to how Stat3-dependent Tfr cells act on Tfh cells compared to Bcl6-dependent Tfr cells. With Bcl6FC mice, we observed increased cytokine production from Tfh cells, whereas this was not observed in Stat3FC mice (data not shown). The increased cytokines made by Tfh cells in Bcl6FC mice likely can explain the altered IgG and IgA responses in these mice. A skewing from IgG to IgA responses may explain the decreased IgG in Bcl6FC mice. Why loss of Tfr cells in Bcl6FC mice, but not in Stat3FC mice, leads to alterations in cytokine production by Tfh cells requires further in-depth study. Overall, Tfh cells appear to be regulated in slightly different ways by Stat3-dependent Tfr cells versus Bcl6-dependent Tfr cells. However, our data are consistent in showing that Tfr cells do not significantly control the proliferation of Tfh and GC B cells in the GC reaction, but rather that Tfr cells regulate the production of antibodies by GC B cells, and that Tfr cells regulate the production of specific Ig isotypes.

Recent reports revealed that Tfr cells derived from Treg cells, and Tfh cells derived from Treg cells or Th17 cells are important for healthy microbiota in the gut, by supporting the production of diverse IgA Abs [24, 25]. However, we did not see any changes of total IgA+ GC B cells in PP from Stat3FC mice. Since Tfr cells in the PP can regulate the diversity of gut microbiota via modulation of antigen specific IgA [38], we evaluated the impact of loss of Tfr cells in PPs on gut microbiota in Stat3FC mice. We collected cecal contents from WT and Stat3FC mice to analyze the gut bacterial composition by 16s rRNA sequencing. However, we were not able to detect a significant difference in bacterial diversity in the gut in Stat3FC mice using this approach (data not shown).

In summary, our study demonstrates the important role of Stat3 in Tfr cell differentiation. We show that Stat3 in Treg cells is required for the suppression of antigen-specific antibody production, whereas Tfr cells do not regulate the sizes of Tfh and GC B cell responses. Further research on the role of Stat3 and Bcl6 in Treg cells on the regulation of humoral responses by Tfr cells is warranted.

## Supporting Information

S1 Fig Basal titers of serum IgM, IgG1, IgG2b, IgG2c and IgG3 are not altered in Stat3CD4KO mice. Serum samples from unimmunized control and Stat3CD4KO mice were collected. IgM, IgG1, IgG2b, IgG2c and IgG3 titers are shown. The X-axis shows the dilution factors. Graphs show mean +/- SEM, n = 3. No significant differences in basal Ig isotype levels were observed by two-way ANOVA.
(TIF)

## References

[1] Breitfeld D, Ohl L, Kremmer E, Ellwart J, Sallusto F, Lipp M, et al Follicular B helper T cells express CXC chemokine receptor 5, localize to B cell follicles, and support immunoglobulin production. The Journal of Experimental Medicine. 2000;192(11):1545–52. Epub 2000/12/06. 11104797 10.1084/jem.192.11.1545 PMC2193094

[2] Schaerli P, Willimann K, Lang AB, Lipp M, Loetscher P, Moser B. CXC chemokine receptor 5 expression defines follicular homing T cells with B cell helper function. The Journal of Experimental Medicine. 2000;192(11):1553–62. Epub 2000/12/06. 11104798 10.1084/jem.192.11.1553 PMC2193097

[3] Zhu J, Paul WE. Peripheral CD4+ T-cell differentiation regulated by networks of cytokines and transcription factors. Immunological reviews. 2010;238(1):247–62. Epub 2010/10/26. 10.1111/j.1600-065X.2010.00951.x 20969597 PMC2975272

[4] Nakayamada S, Poholek AC, Lu KT, Takahashi H, Kato M, Iwata S, et al Type I IFN induces binding of STAT1 to Bcl6: divergent roles of STAT family transcription factors in the T follicular helper cell genetic program. J Immunol. 2014;192(5):2156–66. Epub 2014/02/04. 10.4049/jimmunol.1300675 24489092 PMC3967131

[5] Choi YS, Eto D, Yang JA, Lao C, Crotty S. Cutting Edge: STAT1 Is Required for IL-6-Mediated Bcl6 Induction for Early Follicular Helper Cell Differentiation. J Immunol. 2013 Epub 2013/03/01. 10.4049/jimmunol.1203032 . 23447690 PMC3626564

[6] Ray JP, Marshall HD, Laidlaw BJ, Staron MM, Kaech SM, Craft J. Transcription factor STAT3 and type I interferons are corepressive insulators for differentiation of follicular helper and T helper 1 cells. Immunity. 2014;40(3):367–77. Epub 2014/03/19. 10.1016/j.immuni.2014.02.005 24631156 PMC3992517

[7] Nakayamada S, Kanno Y, Takahashi H, Jankovic D, Lu KT, Johnson TA, et al Early Th1 Cell Differentiation Is Marked by a Tfh Cell-like Transition. Immunity. 2011;35(6):919–31. Epub 2011/12/27. 10.1016/j.immuni.2011.11.012 22195747 PMC3244883

[8] Nurieva RI, Chung Y, Martinez GJ, Yang XO, Tanaka S, Matskevitch TD, et al Bcl6 mediates the development of T follicular helper cells. Science. 2009;325(5943):1001–5. Epub 2009/07/25. 10.1126/science.1176676 19628815 PMC2857334

[9] Vinuesa CG, Cook MC, Angelucci C, Athanasopoulos V, Rui L, Hill KM, et al A RING-type ubiquitin ligase family member required to repress follicular helper T cells and autoimmunity. Nature. 2005;435(7041):452–8. Epub 2005/05/27. 10.1038/nature03555 . 15917799

[10] Linterman MA, Rigby RJ, Wong RK, Yu D, Brink R, Cannons JL, et al Follicular helper T cells are required for systemic autoimmunity. The Journal of Experimental Medicine. 2009;206(3):561–76. Epub 2009/02/18. 10.1084/jem.20081886 19221396 PMC2699132

[11] Odegard JM, Marks BR, DiPlacido LD, Poholek AC, Kono DH, Dong C, et al ICOS-dependent extrafollicular helper T cells elicit IgG production via IL-21 in systemic autoimmunity. The Journal of Experimental Medicine. 2008;205(12):2873–86. Epub 2008/11/05. 10.1084/jem.20080840 18981236 PMC2585848

[12] Hori S, Nomura T, Sakaguchi S. Control of regulatory T cell development by the transcription factor Foxp3. Science. 2003;299(5609):1057–61. Epub 2003/01/11. 10.1126/science.1079490 . 12522256

[13] Fontenot JD, Gavin MA, Rudensky AY. Foxp3 programs the development and function of CD4+CD25+ regulatory T cells. Nature Immunology. 2003;4(4):330–6. Epub 2003/03/04. 10.1038/ni904 . 12612578

[14] Koch MA, Tucker-Heard G, Perdue NR, Killebrew JR, Urdahl KB, Campbell DJ. The transcription factor T-bet controls regulatory T cell homeostasis and function during type 1 inflammation. Nature Immunology. 2009;10(6):595–602. Epub 2009/05/05. 10.1038/ni.1731 19412181 PMC2712126

[15] Zheng Y, Chaudhry A, Kas A, deRoos P, Kim JM, Chu TT, et al Regulatory T-cell suppressor program co-opts transcription factor IRF4 to control T(H)2 responses. Nature. 2009;458(7236):351–6. Epub 2009/02/03. 10.1038/nature07674 19182775 PMC2864791

[16] Chaudhry A, Rudra D, Treuting P, Samstein RM, Liang Y, Kas A, et al CD4+ regulatory T cells control TH17 responses in a Stat3-dependent manner. Science. 2009;326(5955):986–91. Epub 2009/10/03. 10.1126/science.1172702 . 19797626 PMC4408196

[17] Kluger MA, Luig M, Wegscheid C, Goerke B, Paust HJ, Brix SR, et al Stat3 programs Th17-specific regulatory T cells to control GN. Journal of the American Society of Nephrology: JASN. 2014;25(6):1291–302. Epub 2014/02/11. 10.1681/ASN.2013080904 24511136 PMC4033381

[18] Kluger MA, Melderis S, Nosko A, Goerke B, Luig M, Meyer MC, et al Treg17 cells are programmed by Stat3 to suppress Th17 responses in systemic lupus. Kidney International. 2015 Epub 2015/10/16. 10.1038/ki.2015.296 . 26466322

[19] Chung Y, Tanaka S, Chu F, Nurieva RI, Martinez GJ, Rawal S, et al Follicular regulatory T cells expressing Foxp3 and Bcl-6 suppress germinal center reactions. Nature Medicine. 2011;17(8):983–8. Epub 2011/07/26. 10.1038/nm.2426 21785430 PMC3151340

[20] Linterman MA, Pierson W, Lee SK, Kallies A, Kawamoto S, Rayner TF, et al Foxp3+ follicular regulatory T cells control the germinal center response. Nature Medicine. 2011;17(8):975–82. Epub 2011/07/26. 10.1038/nm.2425 21785433 PMC3182542

[21] Wollenberg I, Agua-Doce A, Hernandez A, Almeida C, Oliveira VG, Faro J, et al Regulation of the germinal center reaction by Foxp3+ follicular regulatory T cells. J Immunol. 2011;187(9):4553–60. Epub 2011/10/11. 10.4049/jimmunol.1101328 . 21984700

[22] Ding Y, Li J, Yang P, Luo B, Wu Q, Zajac AJ, et al Interleukin-21 promotes germinal center reaction by skewing the follicular regulatory T cell to follicular helper T cell balance in autoimmune BXD2 mice. Arthritis Rheumatol. 2014;66(9):2601–12. Epub 2014/06/10. 10.1002/art.38735 24909430 PMC4146687

[23] Lu KT, Kanno Y, Cannons JL, Handon R, Bible P, Elkahloun AG, et al Functional and Epigenetic Studies Reveal Multistep Differentiation and Plasticity of In Vitro-Generated and In Vivo-Derived Follicular T Helper Cells. Immunity. 2011 Epub 2011/10/25. 10.1016/j.immuni.2011.07.015 . 22018472 PMC3235706

[24] Kato LM, Kawamoto S, Maruya M, Fagarasan S. Gut TFH and IgA: key players for regulation of bacterial communities and immune homeostasis. Immunology and Cell Biology. 2014;92(1):49–56. Epub 2013/10/09. 10.1038/icb.2013.54 . 24100385

[25] Hirota K, Turner JE, Villa M, Duarte JH, Demengeot J, Steinmetz OM, et al Plasticity of Th17 cells in Peyer's patches is responsible for the induction of T cell-dependent IgA responses. Nature Immunology. 2013;14(4):372–9. Epub 2013/03/12. 10.1038/ni.2552 23475182 PMC3672955

[26] Wu H, Xu LL, Teuscher P, Liu H, Kaplan MH, Dent AL. An Inhibitory Role for the Transcription Factor Stat3 in Controlling IL-4 and Bcl6 Expression in Follicular Helper T Cells. J Immunol. 2015;195(5):2080–9. Epub 2015/07/19. 10.4049/jimmunol.1500335 26188063 PMC4546859

[27] Baumjohann D, Preite S, Reboldi A, Ronchi F, Ansel KM, Lanzavecchia A, et al Persistent antigen and germinal center B cells sustain T follicular helper cell responses and phenotype. Immunity. 2013;38(3):596–605. Epub 2013/03/19. 10.1016/j.immuni.2012.11.020 . 23499493

[28] Wu H, Chen Y, Liu H, Xu LL, Teuscher P, Wang S, et al Follicular regulatory T cells repress cytokine production by follicular helper T cells and optimize IgG responses in mice. European Journal of Immunology. 2016 Epub 2016/02/19. 10.1002/eji.201546094 . 26887860 PMC4896226

[29] Tsuji M, Komatsu N, Kawamoto S, Suzuki K, Kanagawa O, Honjo T, et al Preferential generation of follicular B helper T cells from Foxp3+ T cells in gut Peyer's patches. Science. 2009;323(5920):1488–92. Epub 2009/03/17. 10.1126/science.1169152 . 19286559

[30] Chaudhry A, Rudensky AY. Control of inflammation by integration of environmental cues by regulatory T cells. The Journal of Clinical Investigation. 2013;123(3):939–44. Epub 2013/03/05. 10.1172/JCI57175 23454755 PMC3582113

[31] Ramiscal RR, Vinuesa CG. T-cell subsets in the germinal center. Immunological Reviews. 2013;252(1):146–55. Epub 2013/02/15. 10.1111/imr.12031 . 23405902

[32] Asao H, Okuyama C, Kumaki S, Ishii N, Tsuchiya S, Foster D, et al Cutting edge: the common gamma-chain is an indispensable subunit of the IL-21 receptor complex. J Immunol. 2001;167(1):1–5. Epub 2001/06/22. . 11418623 10.4049/jimmunol.167.1.1

[33] Takeda K, Kaisho T, Yoshida N, Takeda J, Kishimoto T, Akira S. Stat3 activation is responsible for IL-6-dependent T cell proliferation through preventing apoptosis: generation and characterization of T cell-specific Stat3-deficient mice. J Immunol. 1998;161(9):4652–60. Epub 1998/10/30. . 9794394

[34] Raz R, Lee CK, Cannizzaro LA, d'Eustachio P, Levy DE. Essential role of STAT3 for embryonic stem cell pluripotency. Proceedings of the National Academy of Sciences of the United States of America. 1999;96(6):2846–51. Epub 1999/03/17. 10077599 10.1073/pnas.96.6.2846 PMC15857

[35] Reliene R, Schiestl RH. Differences in animal housing facilities and diet may affect study outcomes-a plea for inclusion of such information in publications. DNA Repair. 2006;5(6):651–3. Epub 2006/04/04. 10.1016/j.dnarep.2006.02.001 . 16581314

[36] Park SR, Seo GY, Choi AJ, Stavnezer J, Kim PH. Analysis of transforming growth factor-beta1-induced Ig germ-line gamma2b transcription and its implication for IgA isotype switching. European Journal of Immunology. 2005;35(3):946–56. Epub 2005/02/03. 10.1002/eji.200425848 . 15688346

[37] Hollister K, Chen Y, Wang S, Wu H, Mondal A, Clegg N, et al The role of follicular helper T cells and the germinal center in HIV-1 gp120 DNA prime and gp120 protein boost vaccination. Human Vaccines & Immunotherapeutics. 2014;10(7):1985–92. Epub 2014/11/27. 10.4161/hv.28659 25424808 PMC4186047

[38] Kawamoto S, Maruya M, Kato LM, Suda W, Atarashi K, Doi Y, et al Foxp3(+) T cells regulate immunoglobulin a selection and facilitate diversification of bacterial species responsible for immune homeostasis. Immunity. 2014;41(1):152–65. Epub 2014/07/16. 10.1016/j.immuni.2014.05.016 . 25017466

