# Supplementary figures and images for: Stat3 Is Important for Follicular Regulatory T Cell Differentiation
Source: PLoS One. 2016 May 5;11(5):e0155040. doi: 10.1371/journal.pone.0155040 (PMC4858255; doi:10.1371/journal.pone.0155040)

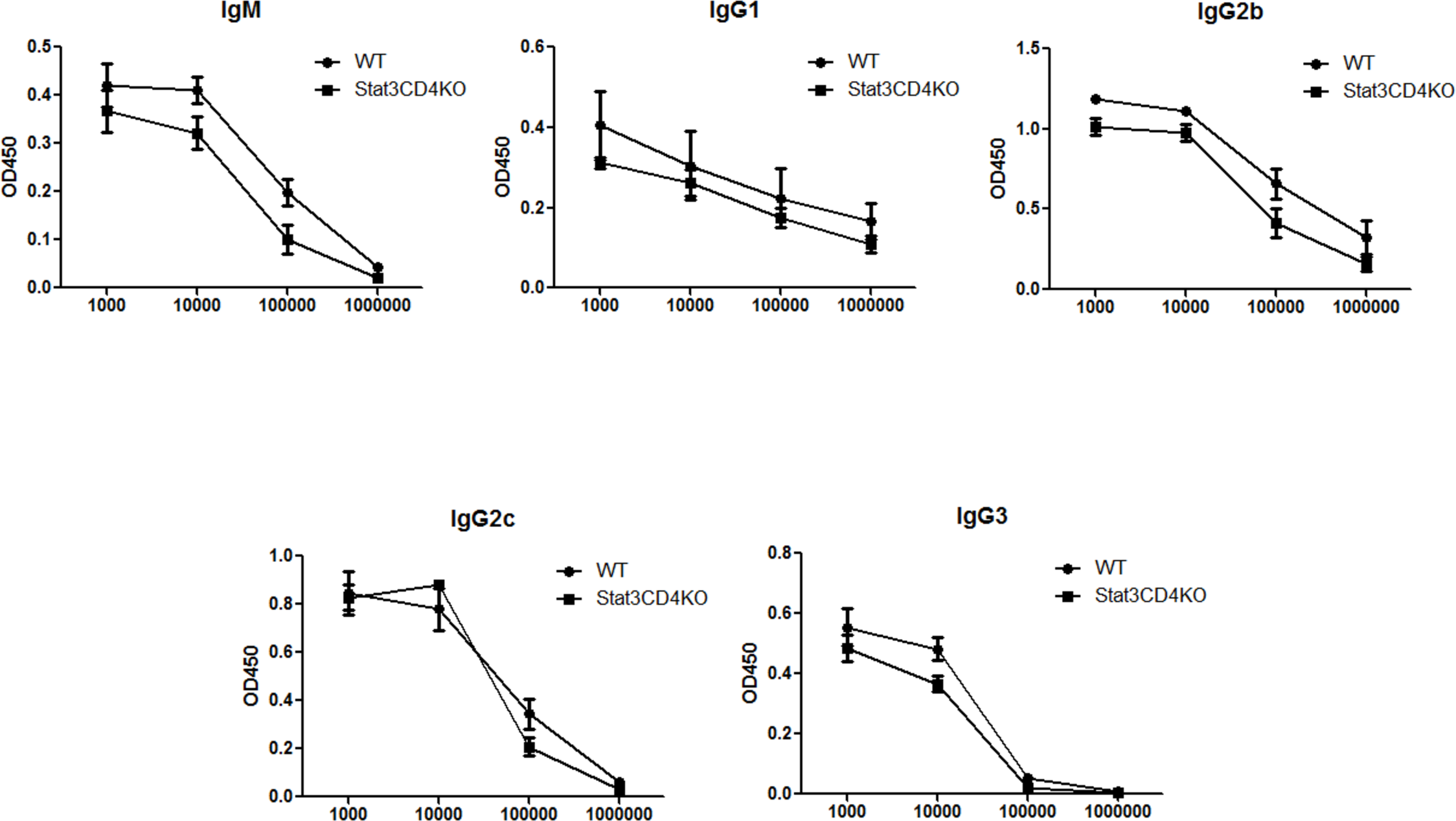

Supplement: S1 Fig — Serum samples from unimmunized control and Stat3CD4KO mice were collected. IgM, IgG1, IgG2b, IgG2c and IgG3 titers are shown. The X-axis shows the dilution factors. Graphs show mean +/- SEM, n = 3. No significant differences in basal Ig isotype levels were observed by two-way ANOVA. (TIF) [file pone.0155040.s001.tif]
